# Generative AI Accelerates Genotype–Phenotype Characterization of a 1600-Case Leigh Syndrome Virtual Cohort from Published Literature

**DOI:** 10.3390/biology15040334

**Published:** 2026-02-14

**Authors:** Lishuang Shen

**Affiliations:** Pathology Informatics, Bioinformatics, and Data Science, Department of Pathology and Laboratory Medicine, Children’s Hospital Los Angeles, Los Angeles, CA 90027, USA; lishen@chla.usc.edu

**Keywords:** generative AI (GenAI), large language model, Leigh disease, Leigh Syndrome Spectrum (LSS), rare disease, human phenotype ontology (HPO)

## Abstract

Leigh Syndrome Spectrum (LSS) is a rare and heterogeneous disease continuum with most published cohorts in small sizes that limit the statistical power. Large-scale meta-analyses with published case-level clinical data extracted from the literature are essential for robust population analysis but are hindered by the burden of manually standardizing the unstructured, heterogeneous, and sparse case-level data from the literature. We developed a novel workflow which is among the first to combine Generative AI (GenAI) with human-in-the-loop curation to overcome this barrier. This pipeline utilized Google’s Gemini-2.5-pro and rapidly processed over 2300 cases from published case data tables in two weeks and achieved >90% accuracy in mapping raw clinical data to Human Phenotype Ontology (HPO) terms. This process rapidly yielded a harmonized LSS virtual cohort of 1679 data-rich cases, which is the largest LSS virtual cohort reported so far, and thus enables characterization of LSS phenotypic and genetic architectures, revealing that autosomal recessive (932 cases) and mitochondrial (752 cases) inheritance are the most common. The most frequently mutated genes were *SURF1* (240 cases), *MT-ATP6* (199), and *MT-ND3* (183). HPO term consolidation identified common hallmark phenotypes, including lactic acidosis, hypotonia, bilateral basal ganglia lesions, and mitochondrial respiratory chain deficiency. The cohort’s scale enabled large-scale survival analysis, revealing that defects in mitochondrial translation are associated with the poorest prognosis (84% mortality in this group) and early onset (0.23 years). Among the deceased group, patients with Complex V mutations were linked to a significantly shorter mean survival time (1.77 years) than those with Complex I (3.70 years) or IV (3.57 years) mutations. This GenAI-driven methodology establishes a scalable framework for rapidly creating analysis-ready virtual cohorts from heterogeneous literature and accelerating population-level study for rare diseases including Leigh Syndrome and other mitochondrial diseases.

## Simple Summary

Leigh Syndrome Spectrum (LSS) is a very rare severe brain disorder, so LSS study is hampered by the small numbers of patients per report and cost in manually merging/standardizing medical data from small reports. This study built a new generative artificial intelligence (GenAI)-based system using Google’s Gemini-2.5-pro and was supervised by medical experts. It rapidly transformed 2300 published patients’ data in just two weeks by converting their unstructured medical information into standardized clinical data dictionaries with over 90% accuracy. This work created one of the largest LSS virtual datasets, with 1679 curated cases. Analysis of this large dataset showed that most patients had either recessively inherited or mitochondrial DNA mutations. The most affected genes were *SURF1*, *MT-ATP6*, and *MT-ND3*. The key symptoms include lactic acidosis, muscle weakness, brain lesions, and mitochondrial dysfunction. The study also revealed that patients with defects in mitochondrial protein production had the worst survival outcomes, with 84% mortality. Patients with Complex V gene mutations survived an average of 1.77 years, half as long as other complexes. This AI-powered approach provides a scalable solution for creating large virtual patient cohorts from the published literature, accelerating research and discovery for Leigh Syndrome and other rare diseases.

## 1. Introduction

Leigh Syndrome (LS, OMIM: 256000, ORPHA: 506) was first described by Denis Leigh in 1951 as Subacute Necrotizing Encephalomyelopathy [1]. This devastating disorder typically manifests before two years of age (early-onset) with complex and variable clinical profiles including developmental delay or regression, hypotonia, ataxia, and brainstem dysfunction [2,3]. Approximately 20% of cases present either in late-onset form in childhood between age 2 and 16, or even rarer, in adult-onset form after 16 years of age [4]. The prognosis for early-onset cases is generally poor, with survival time often limited to only a few years after symptom onset, whereas late-onset cases usually have milder symptoms [4]. LS is the most common pediatric presentation of primary mitochondrial disease (PMD) but remains rare with an estimated prevalence of 1 in 34,000 live births [2]. As clinicians often encounter patients with atypical symptoms or missing characteristic neuroimaging findings for LS such as brain lesions, the term “Leigh-Like Syndrome” (LLS) was adopted. Recognizing the significant clinical and genetic overlap between LS and LLS, the Clinical Genome Resource (ClinGen) Mitochondrial Disease Gene Curation Expert Panel (Mito-GCEP) recently proposed the unifying term “Leigh Syndrome Spectrum (LSS)” to encompass this entire disease continuum [5].

Leigh Syndrome Spectrum (LSS) could be caused by mutations in both the mitochondrial genome (mtDNA) and the nuclear genome [6,7]. Molecular genetic studies have identified over 140 causative genes [5,8]. Most LSS genes encode proteins involved in mitochondrial functions, including the structural subunits and assembly factors of the oxidative phosphorylation (OXPHOS) system, the pyruvate dehydrogenase complex (PDC), and coenzyme Q10 biosynthesis. Additionally, a few transfer RNA (tRNA) genes were reported as causing LSS [5,6,7,8,9].

The existing studies of rare diseases including LSS faced challenges due to their low prevalence, thus the small and geographically dispersed patient cohorts, which limited their statistical power needed for confidently establishing genotype–phenotype correlations, tracking disease progression, identifying prognostic factors, or validating treatment strategies. Meta-analysis of published case data is one proven and commonly used strategy to overcome these cohort size limitations. However, creating a large, comprehensive LSS virtual cohort from published studies has proven to be a tedious and formidable task that is constrained by several critical challenges.

First, published LSS cohorts are typically small in size, ranging from single case reports to just over 200 patients, and are often limited to specific geographic regions or ethnic backgrounds [10,11,12,13,14,15,16,17,18]. In addition, the sources data can be sparse, highly variable in quality, and locked in unstructured formats, including tables, Supplementary Materials, and free-text clinical narratives. These factors mean a big manpower burden to compile a large cohort from published LSS case data. For example, one recent meta-analysis compiled a 385-case virtual cohort from five publications [17], limiting its size and patient representativeness. Our efforts in building a literature-derived registry at MSeqDr Consortium have faced similar challenges in over 7 years of practice [15,19].

A second and more resource-intensive bottleneck is the standardization of clinical data. Phenotypic descriptions across publications are extremely heterogeneous due to inconsistent terminologies, widespread usage of non-standard abbreviations, and free text narrative, so they require expert interpretation to map into controlled vocabularies like the Human Phenotype Ontology (HPO) [20]. Manual curation requires significant time from clinical experts and bioinformaticians which may not always receive appropriate funding and resource support.

To address these bottlenecks, we harnessed the reasoning and natural language processing capabilities of newly emerged Generative AI (GenAI) and Large Language Models (LLMs). Unlike traditional bioinformatics scripts that rely on rigid rules and string semantic matching, GenAI can be instructed through engineered prompts to interpret context, expand abbreviations, and map unstructured text to standardize ontologies, thereby simulating the cognitive capability of a human curator. Recent efforts using GenAI for rare disease include AutoMAxO for extracting Medical Action Ontology treatment terms from abstracts [21], and RAG-HPO, a Python-based tool that leverages retrieval-augmented generation (RAG) to elevate the accuracy of HPO term assignment [22], but these approaches were not designed for rare disease meta-analysis at scale as we are attempting.

In this study, we developed and applied a novel workflow using Google’s Gemini 2.5 Pro, a state-of-the-art GenAI model, to transform case-level data from LSS publications. Our objective was to construct one of the largest-ever LSS virtual cohorts targeting over 2000 published cases, creating a core dataset of cases with rich and harmonized data elements. We focused on standardizing key case data elements essential for meta-analysis, including genetic variants, mode of inheritance (MOI), zygosity, age at onset, age at death, and clinical phenotypes mapped to standard HPO terms. By demonstrating the speed, accuracy, and scalability of this GenAI-driven approach, we aim to establish a new paradigm for rare disease cohort and population study and provide the rare disease community with a toolset and data resource to uncover novel insights into the complex landscape of the Leigh Syndrome Spectrum and other rare diseases.

## 2. Materials and Methods

**Figure 1:**
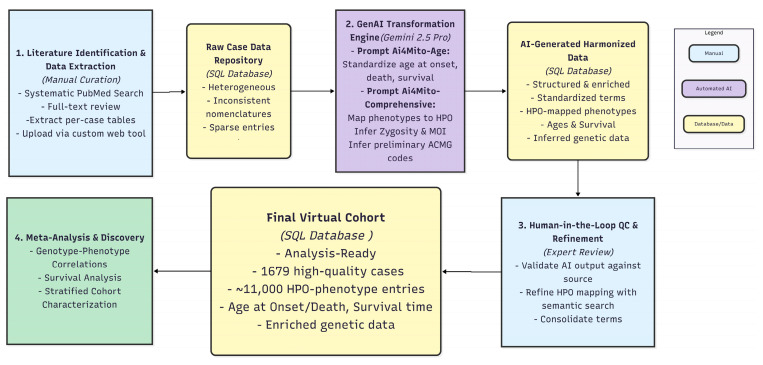
GenAI-powered workflow for virtual cohort creation and curation.

A hybrid framework integrating GenAI and traditional bioinformatics tools was established (Figure 1). The LSS virtual cohort construction started with PubMed search and case data extraction, then the captured case data from the literature underwent GenAI-powered data transformation and human-in-the-loop quality control. Finally, the resulting cohort underwent cohort characterization and survival analysis.

### 2.1. Literature Search and Study Selection

A systematic literature search was conducted in the PubMed/MEDLINE database (from inception to January 2025) to identify studies reporting on Leigh Syndrome Spectrum (LSS) cohorts. The search utilized a combination of terms: (“Leigh syndrome”) AND (“cohort” OR “cases” OR “probands” OR “patients”). Publication abstracts were first screened to identify cohorts of five or more cases. Candidate publications were then subjected to a full-text review to confirm the availability of case-level data in either the main text or Supplementary Materials.

Inclusion criteria for the final cohort required that each case had: (1) a clinical diagnosis consistent with the Leigh Syndrome Spectrum, supported by clinical symptoms, laboratory findings, neuroimaging, or muscle biopsy; (2) genetic analysis identifying causative or candidate variants in mitochondrial DNA (mtDNA) and/or nuclear DNA (nDNA); and (3) detailed case-level data available for extraction.

### 2.2. Data Extraction and Curation

Case-level data includes clinical, demographic, and genomic features, and raw metadata includes PubMed ID, title, the original table name and notes. They are extracted from the publications’ main text or Supplemental Materials. Other saved meta-information includes curator remarks and records of operations in data capture and manipulation, for example, the renaming of conflicting/duplicated column headers and replacing disruptive special characters for compatibility with the web tool below.

Cohort metadata and case-level data were extracted and imported into a relational MySQL (v.5.6) database via a web tool (URL: https://mseqdr.com/vr/vrupload.php, 15 September 2025), as previously described [15], and fully detailed in the associated online documentation for “Virtual Registry Patient Data Loading and Access” (URL: https://mseqdr.org/doc/Virtual_Registry_help.html, 15 September 2025). To manage the high degree of heterogeneity in raw case data reporting, this web-based tool allows curators to upload tabular data from publications regardless of the original column structure or naming conventions. During the import process, the above interactive interface was used for an initial column mapping of raw column headers (e.g., “Pasinetti,” “Mutation”) to a predefined set of standardized core data elements. This initial data harmonization by column mapping is recorded for guiding subsequent data harmonization.

### 2.3. GenAI-Powered Data Transformation and Prompt Engineering

A novel workflow powered by Generative AI (GenAI) (Figure 1) was developed for case data standardization. State-of-the-art LLMs were tested for case data transformation capabilities on a representative case (MS01000020) with complex multiple-value clinical notes. Google Gemini 2.5 Pro [Google AI Gemini API: https://ai.google.com/] was chosen after spot check comparison with other GenAI tools for its satisfactory accuracy, speed, and efficiency in clinical and genomic data transformation. This choice is based on evaluation of multiple open-sourced LLMs (Qwen3, Phi-4) and subscription-based LLMs (Gemini-2.5, ChatGPT-4, and ChatGPT-5).

A structured prompting strategy was developed to guide data harmonization with GenAI. The core prompt assigned GenAI the role of an “AI expert system emulating a board-certified clinical geneticist” and established strict guiding principles to ensure data fidelity, prevent speculation, and maintain a professional tone. Two prompts were engineered for different case data transformation tasks:

Prompt Ai4Mito-Age: This prompt was designed for the extraction and standardization of temporal data, as detailed in the Supplemental Materials. It processed both structured columns and unstructured free-text descriptions to identify and convert per case data for age, age of onset, and age at death into a consistent numerical format (years). It was further instructed to map these values to the corresponding Human Phenotype Ontology (HPO) terms for onset age and death age. When both onset age and death age (or a last known alive status) were available for a case, the prompt calculated the “Survival Time” as a new entry row and provided a rationale (e.g., “Death at age X years, which is Y years since disease onset”). Alternatively, if the patient is alive status, GenAI was instructed to state the Survival Time as “Alive at age x years, which is y years since disease onset age”.

Prompt Ai4Mito-Comprehensive: This prompt orchestrated a multi-step workflow for the comprehensive harmonization of clinical, demographic, and genomic data. It was instructed to parse unstructured phenotypic narratives, disaggregate them into individual clinical features, and map each phenotype feature to the most specific HPO term. Gene, inheritance mode, and zygosity information are also mapped from the input or inferred. Raw data is mapped to the full terms with reference to the per cohort meta data including the abbreviation keys and the curator enforced column mapping (Tables S1 and S2). For LSS-specific diagnoses, the LLM model was guided by the diagnosis criteria as established by the ClinGen Mito-GCEP expert pane [5].

### 2.4. Human-in-the-Loop Quality Control and Phenotype Data Consolidation

All data generated using GenAI underwent a multi-stage quality control (QC) and consolidation process. Within the human-in-the-loop framework (Figure 1), an initial manual review was performed by a domain expert to verify the fidelity of the AI’s output against the source data and HPO dictionary. Systematic consolidation was then performed within the SQL database to harmonize synonymous data elements (e.g., ‘Gene,’ ‘Gene Symbol’ were consolidated to ‘GeneName’).

The accuracy of GenAI-inferred HPO term mapping was rigorously evaluated. Initially, GenAI-mapped terms were validated against the official HPO dictionary. If for a GenAI-derived term, both HPO term name-based mapping and HPO ID-based mapping were not directly matched to the HPO dictionary, a custom bioinformatics web tool, HPO Annotator [19] within Phenotype Workbench (https://mseqdr.org/clinical/pa.php, 15 September 2025), was employed to perform a semantic similarity search in the batch, which ranks HPO term candidates based on a composite score calculated from the semantic similarity to the HPO term names, synonyms, and definitions. Following this, a final HPO terms selection was made through expert review within this web tool. The selected mappings were then saved to the MySQL relational database, where the corresponding cases are annotated with the newly picked HPO terms.

Ontology-guided phenotype data consolidation was conducted to overcome the heterogenous specificity levels among published raw case phenotypes and their mapped HPO terms. To facilitate a high-level meta-analysis and align with established LSS diagnostic criteria, the mapped HPO terms, which exist at various levels of specificity within the ontology’s hierarchical tree structure, were consolidated into major phenotypic groups. This was achieved by leveraging the ontological relationships inherent in the HPO graph path. Specific granular phenotypes (child terms) were systematically mapped to their higher-level ancestor terms (parent groups) seen in the cohort if they fell within a predefined ontology path with a distance between 0 levels (means self-to-self) and up to 20 levels. The resulting major “umbrella” phenotype groups were then validated against clinically recognized categories used in LSS diagnosis [5]. For each major group, we estimated the total number of constituent child terms and the number of unique cases exhibiting at least one phenotype within that category. The saved results for each umbrella phenotype group include HPO names of all the child terms with cases numbers carrying each child terms.

### 2.5. Cohort Characterization and Statistical Analysis

Statistical analyses were performed using R software (v.4.1, R Core Team, 2024). Clinical features, age at onset, age at death, and survival time were compared across different patient strata defined by MOI, sex, genetic locus (mtDNA vs. nDNA), and specific causative genes. Group differences for numeric variables were assessed using a Student’s *t*-test or ANOVA, while Fisher’s Exact Test was used for categorical variables. A two-tailed *p*-value ≤ 0.05 was considered statistically significant. HPO terms from cohort cases were visualized with R package ontologyPlot (v.1.7.0). Kaplan–Meier survival analysis and visualization were carried out with R survival (v.3.8.3), survminer (v.0.5.1), and tidyverse (v.2.0.0) packages.

## 3. Results

### 3.1. Construction of the Leigh Syndrome Spectrum Virtual Cohort

The literature search of PubMed and a manual review identified 38 publications that met the inclusion criteria with tabular, case-level data suitable for extraction. Studies were excluded if they did not provide case-level data in a tabular format within the main text or Supplementary Materials. The case-level data from qualifying publications were subsequently imported into a local MySQL database using a custom-developed web tool, which also supported the initial mapping of raw table headers to standardized core data elements (Figure 1).

The merged dataset was converted into a case-by-feature matrix, comprising 2314 LSS cases from 38 publications with individual published cohort sizes ranging from 5 to 275 cases (Table 1 for the top 10 publications, Table S3 for the full publication list). These cases’ raw data underwent GenAI standardization and human-in-the-loop post-AI curation to transform data for the core data elements: Age_at_Onset, Age_at_Death, DIAGNOSIS, GENE, GENOTYPE (Zygocity), MODE_OF_INHERITANCE, PHENOTYPE, VARIANT_cDNA, VARIANT_mtDNA, and VARIANT_Protein.

**Figure 2:**
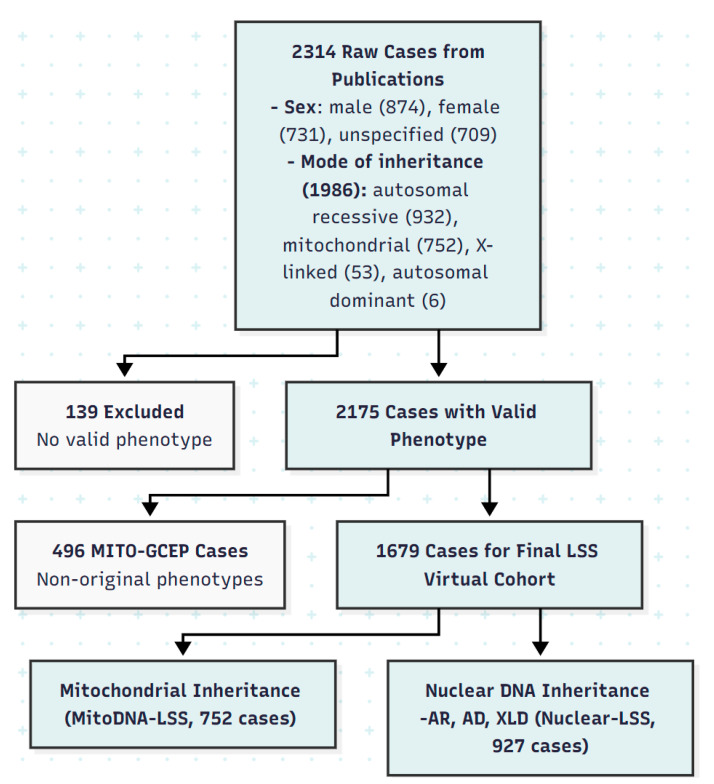
Flowchart of LSS virtual cohort construction.

After GenAI standardization and expert curation, the raw cohort comprised 2314 cases (Figure 2). Of the 2314 cases, 1605 patients had sex information available in the raw data: 874 (54.5%) male, 731 (45.5%) female, 709 unspecified (Table 2). Following phenotype data mapping validation, 2175 cases with valid phenotype information were retained. Cases from PubMed 37255483 [5] were subsequently excluded, as their phenotypes had been previously transformed by ClinGen Mito-GCEP members from original publications, thus no longer representing raw phenotypes eligible for AI-based transformation in this study. The final literature-derived LSS virtual cohort consisted of 1679 cases, which were used for meta-analysis.

### 3.2. GenAI-Powered Harmonization of Heterogeneous Data Elements

A primary challenge in this rare disease meta-analysis was the extreme data heterogeneity across cohorts. This heterogeneity manifested primarily in two ways: (1) as inconsistent data field naming (with dozens of raw terms for a single data element) and (2) as inconsistent formats and nomenclatures for phenotype and genomic values (Table S1). For example, the raw LSS dataset contained 48 different column headers related to phenotypes (Table S1), plus highly complex and inconsistent value encoding. The case data heterogeneity across cohorts resulted in a sparse and inconsistent case-feature matrix that required extensive normalization before statistical analysis could be performed, so standardizing clinical phenotypes is a cornerstone of virtual cohort construction.

Ai4Mito-Comprehensive is the GenAI prompt for instructing LLM to standardize the raw case data, including mapping clinical phenotypes to HPO terms. It instructs GenAI to perform a multi-step process: (1) identify all phenotype-related data; (2) expand abbreviations using the abbreviation key input, the context from the publication, and GenAI’s own knowledge base; (3) disaggregate multi-symptom entries into individual phenotypes; (4) optionally, infer phenotype terms from biochemical data (e.g., classifying high lactate value number as “Elevated CSF lactate” by comparing with the normal range within LLM knowledge); (5) map the standardized phenotype to the most appropriate HPO term names and HPO IDs; and (6) generate a structured tabular output, including rationales in mapping process (Figure 1, Table 3 and Table S1).

**Figure 3:**
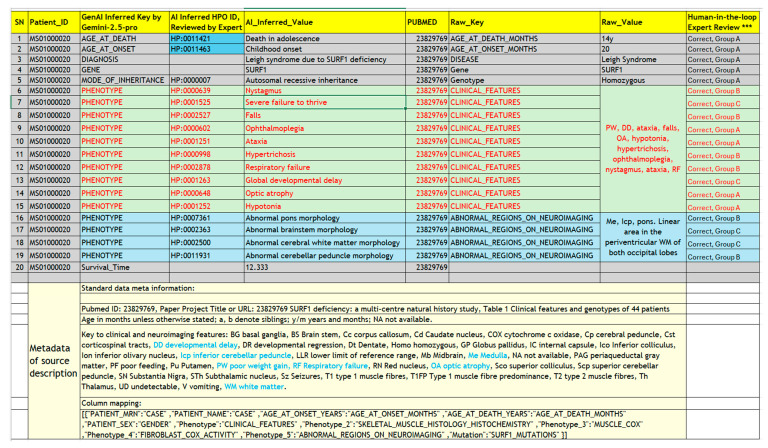
GenAI maps raw phenotypes and abbreviation into HPO terms (Case MS01000020). Figure 3 note: *** Validation Group A, B, C are defined as in Table 5.

To select an optimal LLM model, we performed a comparative evaluation using a representative complex case (Patient ID: MS01000020, Figure 3, and URL https://mseqdr.com/vr/caserecord.php?set=demo&type=cpmuid&cpmuid=MS01000020, 15 September 2025), whose complicated phenotype profile was captured in two unstructured, multi-symptom strings using abbreviations:

(a) CLINICAL_FEATURES = “PW, DD, ataxia, falls, OA, hypotonia, hypertrichosis, ophthalmoplegia, nystagmus, ataxia, RF” and,
(b) ABNORMAL_REGIONS_ON_NEUROIMAGING = “Me, Icp, pons. Linear area in the periventricular WM of both occipital lobes”.

Gemini 2.5 Pro successfully disaggregated the raw text with the guidance from provided abbreviation keys and correctly mapped all 14 identifiable phenotypes to their corresponding HPO full terms and IDs (Figure 3, Table S5). In contrast, while ChatGPT-5 also identified 14 terms, two of the inferred HPO IDs were invalid (non-existent) in the HPO dictionary—a sign of hallucination (Table S6). ChatGPT-5 also failed to correctly standardize age at disease onset and death into HPO IDs, whereas Gemini-2.5-Pro inferred both correctly. The locally installed open-source models showed significant limitations in testing, for instance. For example, Phi-4-mini failed to disaggregate the multi-symptom entries, while Qwen3-30B-A3B-Q4-K_M correctly identified only three HPO IDs out of twelve inferred HPO IDs (Tables S7 and S8).

Based on this preliminary evaluation, it was evident that most tested LLMs underperformed in phenotype-to-HPO mapping accuracy compared to Gemini 2.5 Pro. As the primary goal of this study was rapid cohort construction for meta-analysis rather than a comprehensive LLM benchmark, we did not pursue further testing with the underperforming models. Consequently, Google’s Gemini 2.5 Pro was selected as the primary engine for clinical data transformation workflow due to its superior initial accuracy in clinical text-to-HPO mapping, processing speed, and ability to adhere to structured output formats. Further efforts focused on optimizing prompts for Gemini and establishing a robust human-in-the-loop validation process to maximize both speed and final data quality.

Practical scalability was another critical factor in the model selection. Gemini 2.5 Pro had the largest context window (up to 1 million tokens) and high-throughput capacity via its AI Studio interface (https://aistudio.google.com/), both of which were essential for processing our large dataset of over 2000 cases. In practice, its server speed allowed for the processing of up to 40,000 input tokens in a single session without timeout error. In contrast, other models were limited by smaller context windows (e.g., 32 k tokens on ChatGPT-5 web interface) and more restrictive daily usage quotas, rendering them unsuitable for the scalability demands of this project.

Age-related data was another ideal standardization target for GenAI automation to overcome the high variability in raw data formats (e.g., years, months, days, life stages) (Table 2 and Table S1). The Ai4Mito-Age prompt was designed to instruct the LLM in mapping dozens of raw age columns to standard terms (Age, Age_of_Onset, Age_at_Death), converting values to a numerical format in years (e.g., “Alive (10y3m)” to 10.25), and mapping these values to the corresponding HPO terms for onset and death stages. GenAI correctly inferred age information from both tabular data and unstructured free-text clinical notes.

Downstream manual quality control of 2341 GenAI-mapped onset age entries identified 90 entries for corrections, an error rate of only 3.8%. These corrections primarily involved reclassifying missing age data being initially miscoded as “0”. Another correction was revising the HPO term for “Late onset” to match the traditional LSS-specific definition (≥2 years) that LSS publications used.

Finally, standardizing age data enabled the calculation of survival time for 770 cases where both onset and death/alive ages were standardized to numerical values in years. A total of 399 cases had definitive ages at death to calculate their final survival time, and another 371 cases had no death age reported so their minimum survival duration was calculated based on their last alive age at the time of reporting. 

### 3.3. Enrichment of Genetic Data Elements Through GenAI Reasoning

GenAI’s reasoning capabilities were utilized to enrich the genetic dataset by inferring from the implicit contextual case data. The MOI was inferred and standardized to HPO terms for 1986 cases (85.7%), representing a 2.97-fold increase over the 685 cases where MOI was explicitly stated in the raw data. And 89.4% of the final MOI entries were GenAI-inferred (Table 4). Similarly, Genotype/zygosity (e.g., homozygous, heterozygous) was standardized for 832 cases, a 4.7-fold increase from the 176 cases in the original dataset (Table 4). This enrichment was achieved by instructing the AI to interpret implicit information within the input (clinical notes, gene, disease, paper title information, etc.), to cross-reference with its foundational knowledge of gene-disease associations, and to utilize real-time web content to infer genotype and MOI information.

Leveraging its reasoning capabilities, the GenAI model assigned standardized HUGO Gene Nomenclature Committee (HGNC) gene symbols to 68% of cases. In addition to processing explicit gene names, it accurately inferred genes from associated data points such as HGVS nomenclature, transcript information, and the broader context of the source publication. The accuracy of these gene mappings was confirmed to be high upon validation against the HGNC database.

### 3.4. Human-in-the-Loop Validation of GenAI-Generated Phenotype Mappings

For validation and quality control, all GenAI-generated phenotype mappings underwent expert review through comparison with HPO term IDs and names in the HPO dictionary. HPO mapping comparison patterns were categorized into three validation groups based on the patterns of concordance between the AI-inferred HPO term against the HPO name, synonym, and ID in the ontology dictionary (Figure 3, Table 5). Validation Group A represented the highest quality mappings, where the GenAI correctly identified both the HPO term name (or an exact synonym) and its corresponding HPO ID. Group B consisted of entries where the GenAI correctly identified the valid HPO term name or synonym, but assigned an incorrect or non-existent HPO ID (hallucination). Group C comprised entries where the AI output did not directly match an official HPO term name and synonym, nor HPO ID, thus requiring sematic match using the web-based HPO Annotator.

**Table 5:** Validation of GenAI-inferred phenotype to HPO mappings.

Validation Group	Description	Cases (n)	Source Publications	Phenotype Entries (n)	% of Total Entries
Group A	Exact Match: Correct HPO Term Name & Correct ID	1668	31	9270	65.80%
Group A.2	Synonym Match: Correct HPO Synonym & Correct ID	57	7	65	0.50%
Group B	ID Hallucination: Correct HPO Term/Synonym but Incorrect ID	925	33	1381	9.80%
Group C	No Direct Match: Requires Semantic Search	1683	38	3378	24.00%
Total		N/A *		14,094	100%

* Note: Case counts do not sum to the total cohort size because a single patient often presents with multiple phenotypes falling into different validation groups. Table Legend: (a) Validation Group A (Verified by Direct Match): The GenAI output matched the official HPO term name and ID perfectly. No manual correction required. (b) Validation Group A.2 (Verified by Synonym Match): The GenAI output matched a recognized HPO synonym and provided the correct corresponding ID. No manual correction required. (c) Validation Group B (ID Correction Required): The GenAI correctly identified the clinical concept by name or synonym but generated an incorrect or non-existent HPO ID. These required manual ID correction. (d) Validation Group C (Semantic Mapping Required): The GenAI output did not match the HPO name or synonym directly, but the context is relevant. These entries required processing via the HPO phenotype tool to identify the closest semantic match.

Human-in-the-loop review of approximately 14,000 GenAI-derived case by phenotype entries confirmed that the GenAI achieved a high degree of accuracy in HPO mapping. In 77.3% of phenotype entries (Groups A and B combined), the model correctly identified the appropriate HPO term name or a valid synonym (Table 5). However, for Group B, manual correction was required to rectify the hallucinated HPO IDs.

The remaining 3378 entries (Group C) were often contextually relevant to the raw phenotype but failed direct string matching against the HPO dictionary. To resolve this, these terms were processed using the HPO Annotator, a web-based semantic similarity search tool (URL: https://mseqdr.org/clinical/pa.php, 15 September 2025). This tool ranked potential HPO matches based on semantic similarity for expert selection [19]. This semi-automated curation step successfully rescued an additional 2860 entries. Consequently, the hybrid GenAI–human workflow resulted in 91% of all extracted phenotype entries being reliably mapped to standardized HPO terms, establishing a deeply characterized dataset for downstream meta-analysis.

### 3.5. HPO-Guided Consolidation of Phenotype Data

**Figure 4:**
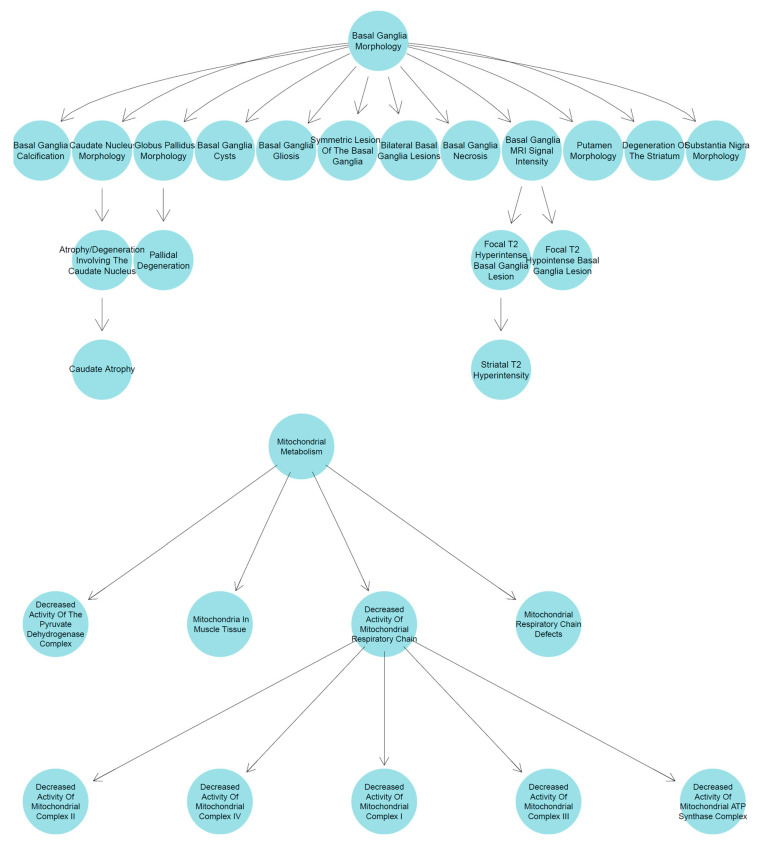
HPO-Based case phenotype consolidation for abnormal basal ganglia morphology (HP:0002134, (Upper)) and abnormality of mitochondrial metabolism (HP:0003287, (Bottom)).

To enable a high-level meta-analysis and align with established LSS diagnostic criteria, the mapped HPO terms, which existed at various levels of specificity across the published studies (Figure 4), were consolidated into major phenotypic groups.

The initial raw dataset contained 2058 cases with 2787 unique raw phenotype descriptors. Because many of these descriptors were multi-symptom lists rather than single entries, they initially formed only 7472 case-by-phenotype combinations. Following the GenAI-driven mapping and subsequent manual curation, the dataset was expanded to approximately 14,000 case phenotype entries, comprising 938 unique HPO terms. This increase in entries is a direct result of the AI’s successful disaggregation of the multi-value raw phenotype lists into discrete, standardized data points.

Phenotype term consolidation was achieved by systematically traversing the HPO’s hierarchical graph structure (Figure 4). From the 938 unique HPO terms in the cohort, we identified 252 “parent” terms that subsumed more specific descendant terms also present in our data. A total of 82 of the terms are at the top levels within the cohort’s HPO terms (Table S4). The major umbrella phenotype groups are picked against LSS diagnosis criteria [6] and detailed in the clinical characterization sections below. This approach consolidated numerous more specific phenotypes under clinically relevant parent terms. For example, the umbrella group ‘Abnormality of brain morphology’ (HP:0012443) consolidated 95 distinct, more granular HPO terms present in the cohort (Table S4), and one of its child terms, ‘Abnormal basal ganglia morphology’ (HP:0002134), has 19 child terms that are up to three levels more granular (Figure 4, Table S4). Similarly, ‘Abnormality of the eye’ (HP:0000478) and ‘Abnormality of movement’ (HP:0100022) subsumed 86 and 61 descendant terms, respectively.

This ontological consolidation reduced the dimensionality of the phenotype data, thereby enabling robust, well-powered statistical comparisons across major clinical characteristics relevant to LSS.

### 3.6. Cohort Demographics and Genetic Spectrums

#### 3.6.1. Overview

Following data extraction, GenAI-powered harmonization, and human-in-the-loop refinement, a final core dataset of 1679 high-quality cases was established from an initial pool of 2314 cases identified in the literature (Figure 2). This large-scale virtual cohort enables meta-analysis with unprecedented statistical power.

In our survival analyses, we addressed missing outcome data by including only the subset of 704 patients for whom a definitive outcome (age at death) or a last known follow-up age was explicitly available. Individuals who were alive at the time of their report were treated as right-censored data points.

**Figure 5:**
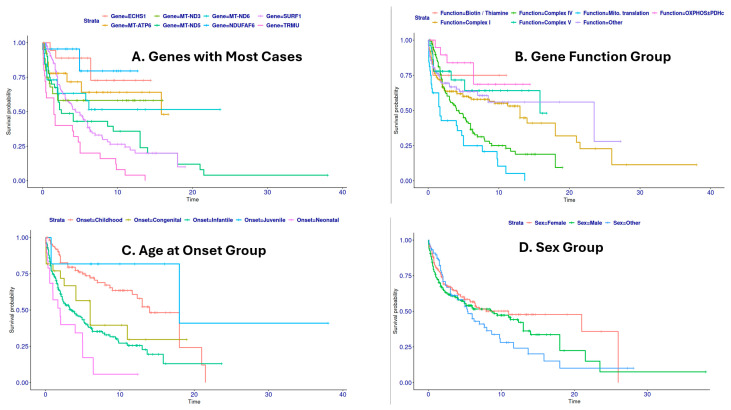
Kaplan–Meier survival analysis and visualization.

Of the 1605 patients with available sex information (Table 2), there was a male predominance (874 males, 54.5%; 731 females, 45.5%, Chisq Test *p*-value 0.0004). A preliminary survival analysis suggested a potential sex-based difference in outcomes: the proportion of deceased males (11.2%) was higher than that of females (9.4%) but with a non-significant Fisher’s Exact Test *p*-value 0.42. Conversely, a higher percentage of female patients had a known “alive” status at the time of reporting (10.8% vs. 7.8% for males). While these data suggest a possible increased risk and poorer survival for males in LSS, definitive conclusions are limited by the uncontrolled nature of a literature-derived cohort. There is no significant difference in the survival time between males and females (*t*-test *p*-value = 0.92). The survival analysis reveals that though females and males have similar onset and survival trend in the early ages, the females show improved 10-year survival, though the data is limited (Figure 5D).

The GenAI workflow successfully inferred MOI for 1986 cases (85.9% of the total cohort), a nearly threefold increase over the 685 cases where it was explicitly stated. The most common inheritance pattern was autosomal recessive (932 cases), followed by mitochondrial (752 cases), X-linked (53 cases), and autosomal dominant (6 cases). Males (26 cases) are over-represented compared to females (8 cases) for cases with X-linked inheritance, but the mean age at onset are similar at 2.33 and 2.11 years, respectively. These X-linked cases were pooled from 12 publications to reach the size.

#### 3.6.2. Age at Onset, Death, and Survival Analysis

Age at onset data was standardized for 1684 patients. The disease predominantly manifests in early life, with Infantile onset (28 days to 1 year; 41.0%) and Childhood onset (1 to 5 years; 34.3%) being the most common HPO disease onset categories. Less frequent were Juvenile onset (12.2%), Neonatal onset (4.5%), and Congenital onset (4.3%). Adult-onset LSS (>16 years) was rare, accounting for only 3.7% of cases (Table 6).

According to classical LSS onset term definition, which is different from HPO terms, 70.8% of patients (1192 of 1684) were early-onset LSS (≤2 years of age), 25.5% (430) were late onset (from 2 years to 16 years old), and 3.7% (62) were “adult onset” (after 16 years age). The mean age of disease onset for the cohort was 2.03 years.

For the 280 patients with a recorded age at death, the median age of onset was 0.97 years, and the median age at death was 2.08 years. The median survival time from onset to death was 2.4 years.

Antenatal onset is associated with the worst survival, followed by the Neonatal and Congenital onset groups. They have a 5-year survival rate of 0.5 or much lower. On the contrary, Childhood onset and Juvenile onset are associated with much improved 5-year survival rate (Figure 5C).

#### 3.6.3. Genetic Spectrum of the LSS Virtual Cohort

Genetic etiology was identified in 1460 cases, split between nuclear DNA (nDNA) mutations (830 cases) and mitochondrial DNA (mtDNA) mutations (630 cases).

The most frequently implicated nuclear gene was *SURF1*, identified in 240 cases and associated with complex IV deficiency. Other common nuclear genes included *NDUFAF6* (33 cases), *ECHS1* (32 cases), and *PDHA1* (31 cases).

Among mtDNA-encoded genes, *MT-ATP6* (199 cases), *MT-ND3* (183 cases), *MT-ND5* (124 cases), and *MT-ND6* (90 cases) have the most pathogenic variants (mutations). Four specific mtDNA variants accounted for a significant portion of these cases: m.8993T>G (*MT-ATP6*, 101 cases), m.13513G>A (*MT-ND5*, 79 cases), m.10197G>A (*MT-ND3*, 78 cases), and m.10191T>C (*MT-ND3*, 56 cases).

Kaplan–Meier survival analysis reveals that *TRMU* mutations are associated with the worst prognosis (Figure 5A). In addition, among the 28 cases with *TRMU* mutations, all three individuals carrying the c.2T>A variant exhibited a significantly shorter survival time of no more than four months. *SURF1* and *MT-ND5* have similar and better survival curves, especially during the first few years after disease onset, whereas *ECHS1* and *NDUFAF6* mutations are associated with best survival. So, the large-scale virtual cohort enabled the identification of interesting genotype–survival correlations thanks to the increased power.

#### 3.6.4. Age at Onset and Survival Time Vary by Mitochondrial Biochemical Function Group

To investigate the relationship between biochemical defects and clinical outcomes, we analyzed a subset of 704 cases for which both age of onset and an outcome (age at death or alive age at last follow-up) were available, allowing for the estimation of exact survival time. For subgroup analysis, these cases were stratified by their pathogenic mutations’ affected genes, and then the genes served as the proxy in mapping to the mitochondrial biochemical function group (Table 7).

The most prevalent mutations within this sub-cohort were related to Complex I, Complex IV, Complex V, and mitochondrial translation. Significant differences in age of onset and survival time were observed among these groups (Figure 5B, Table 7). Based on the Kaplan–Meier survival analysis, mitochondrial translation is associated with the poorest survival and earlier onset, closely followed by Complex V defects (Figure 5B).

Mutations (defect) in mitochondrial translation were associated with the likely poorer prognosis, comprising 26 (84%) deceased patients compared to only five surviving patients (16%). This group had the earliest mean age of onset (0.23 years; Congenital/Neonatal), yet the mean survival time for deceased patients (2.99 years) was comparable to that of the Complex I and IV groups.

Patients with Complex I and Complex IV mutations (defect) represented the largest subgroups and were present in similar proportions in both the deceased and surviving cohorts. Their survival curves largely overlap (Figure 5A). Among deceased patients, both groups had a mean age of onset under one year (Infantile or Neonatal Onset) and comparable survival times. In the surviving cohort, however, the Complex I group had a later mean onset (2.03 years) and a longer observed survival duration (7.3 years). In contrast, the surviving Complex IV group had an earlier mean onset (1.4 years) and a shorter observed survival duration (3.86 years).

The Complex V mutations (defect) group included 14 deceased and 31 surviving patients. Deceased patients with Complex Mutations (defect) had a later mean age of onset (1.65 years; Childhood onset) but a markedly shorter mean survival time of 1.77 years, approximately half that observed for Complex I (3.70 years) and Complex IV (3.57 years).

The “OXPHOS ± PDHc enzyme” group consisted of 14 surviving patients with an early onset (mean 0.6 years) and the longest observed survival (mean 8.17 years), and three deceased patients with a very early onset (mean 0.25 years) and a survival time of 3.05 years. The best survival pattern is evident from the survival curve (Figure 5B).

Finally, the “Biotin/Thiamine” group included only four patients, all of whom were in the surviving cohort. They had an early onset (mean 0.24 years) and a longer observed survival of 6.07 years. However, the small sample size in this group precludes definitive conclusions.

### 3.7. Clinical Characteristics of the LSS Virtual Cohort

#### 3.7.1. Overview of Major Phenotypic Groups

Analysis of the harmonized virtual cohort revealed the prevalence of major clinical features based on the HPO-consolidated phenotype groups. For the 1679 core cohort cases, there are 11,000 case-by-phenotype entries, with phenotypes per case ranging from 0 to 30. Among them, 1576 cases (94%) had valid HPO terms mapped after GeneAI mapping and human validation, where each case has from 1 to 29 HPO terms (median = 4, mean = 6.26, SD = ±5.66; 25th and 75th percentiles at 2 and 9, respectively). The four most frequently reported clinical sign categories, which are central to LSS diagnostic criteria, were abnormality of brain morphology (HP:0012443), present in 963 cases; neurodevelopmental abnormality (HP:0012759), reported in 894 cases; acidosis (HP:0001941), documented in 794 cases; and decreased activity of mitochondrial respiratory chain (HP:0008972), reported in 613 cases.

#### 3.7.2. Lactate Abnormalities and Acidosis

Acidosis was a prominent biochemical feature, with Lactic acidosis (HP:0003128) being the most common subtype, affecting 654 patients. This umbrella term consolidated several specific descriptors, including persistent, congenital, severe, and chronic lactic acidosis. Elevated lactate was also frequently documented in specific tissues: Increased CSF lactate (HP:0002490) was reported in 237 cases, and elevated brain lactate by MRS (HP:0012707) was noted in 83 cases. Furthermore, increased circulating lactate concentration (HP:0002151) was documented in 136 patients, and metabolic acidosis (HP:0001942) was specified in 45 cases.

#### 3.7.3. Neuroimaging Findings

Neuroimaging abnormalities were a hallmark of the cohort and for LSS diagnosis. Abnormal basal ganglia morphology (HP:0002134) was the most common finding, reported in 653 cases. This category encompassed 18 distinct child HPO terms, with the most frequent being bilateral basal ganglia lesions (HP:0007146, 289 cases) and abnormal basal ganglia MRI signal intensity (HP:0012751, 248 cases). Abnormal brainstem morphology (HP:0002363) was documented in 358 cases, most commonly as abnormal brainstem MRI signal intensity (HP:0012747, 203 cases). Finally, abnormal cerebral white matter morphology (HP:0002500) was reported in 150 cases.

#### 3.7.4. Neuromuscular and Movement Abnormalities

Neuromuscular dysfunction was highly prevalent. Abnormal muscle tone (HP:0003808) was described in 670 patients, with hypotonia (HP:0001252) being the most prominent feature (502 cases), followed by hypertonia (HP:0001276, 75 cases) and spasticity (HP:0001257, 65 cases). Muscle weakness (HP:0001324) was reported in 250 cases.

Movement disorders, categorized under abnormality of movement (HP:0100022), were present in 473 patients and most frequently included dystonia (HP:0001332, 154 cases), tremor (HP:0001337, 45 cases), gait ataxia (HP:0002066, 29 cases), hyperreflexia (HP:0001347, 41 cases), and myoclonus (HP:0001336, 37 cases).

#### 3.7.5. Developmental, Ocular, and Other Systemic Manifestations

Neurodevelopmental abnormality (HP:0012759) was a core feature, affecting 894 cases. The most common sub-category was neurodevelopmental delay (HP:0012758), reported in 699 patients, which included global developmental delay (HP:0001263, 596 cases) and developmental regression (HP:0002376, 305 cases).

Ocular abnormalities were also frequent, with abnormality of the eye (HP:0000478) documented in 559 cases. The most common descendent term findings were nystagmus (HP:0000639, 162 cases), ophthalmoplegia (HP:0000602, 135 cases), ptosis (HP:0000508, 98 cases), and optic atrophy (HP:0000648, 75 cases).

Seizures (HP:0001250) were reported in 351 patients, with specific seizure types including generalized-onset, infantile spasms, and focal-onset seizures.

Abnormalities of the respiratory system (HP:0002086) were noted in 296 cases, with respiratory insufficiency (HP:0002093) being the most common manifestation (156 cases), often progressing to respiratory failure (HP:0002878, 81 cases).

#### 3.7.6. Biochemical Measure of Mitochondrial Respiratory Chain Activity

A decreased activity of mitochondrial respiratory chain (HP:0008972) is reported biochemically for 613 cases, associated with the following 5 child HPO terms related to five mitochondrial complexes’ deficiency (Figure 4, bottom panel).

Complex I deficiency (HP:0011923, 425 cases), Complex IV deficiency (HP:0008347, 150 cases), Complex III deficiency (HP:0011924, 14 cases), mitochondrial ATP synthase Complex deficiency (complex V, HP:0011925, 13 cases), and Complex II deficiency (HP:0008314, two cases). It is noted that there are 185 cases (11.0%) carrying mutations in genes from Complex V and more than the 13 cases here with explicitly reported phenotype deficiency.

## 4. Discussion

To the best of our knowledge, this is the first report to harness generative AI for comprehensive transformation of literature-derived, case-level data in rare diseases. Using this approach, we quickly constructed the largest-ever virtual LSS cohort to date comprising 1679 cases, which is more than four times larger than the previous largest virtual cohorts of 275 cases [16] and 385 cases [17]. This virtual cohort complements traditional LSS cohorts built through active patient enrollment which are limited in size and thus statistical power. Meta-analysis with large-scale and harmonized virtual cohorts is an important tool for studying rare diseases genetics, disease courses, and etiology. Historically, rare disease meta-analysis often uses aggregated summary statistics from individual cohorts through mathematical calculations but is unable to directly work on case-level data due to the profound technical challenges and resource-intensive manual curation required to harmonize heterogeneous data. In the authors’ previous attempt at building a mitochondrial disease registry from published cases [15], the effort to merge and harmonize data at the case-level has been technically challenging and hampered by the heavy manpower burden in manually compiling and curating the highly heterogeneous case-level clinical data from the published literature. This study demonstrates a paradigm shift in this process, leveraging on a human-in-the-loop Generative AI pipeline to rapidly construct and characterize large virtual cohorts, thus enabling a more robust clinical characterization analysis of rare diseases.

### 4.1. The Generative AI-Enabled Literature Mining Pipeline: A Technical Perspective

#### 4.1.1. Advantages of the GenAI Workflow

The primary advantages of our Generative AI-Enabled Literature Mining Pipeline are speed and scalability. The process of extracting, standardizing, and harmonizing data from over 2300 raw cases into the largest-ever LSS cohort was completed in approximately two weeks. Based on author’s experience, completing this process using traditional methods would have required months of manual work by skilled biocurators and domain experts. Another key advantage is data enrichment of implicit data through AI inference. The GenAI model extends beyond merely standardizing explicitly provided case data by inferring data points “missing” from the whole context of input for a case, the LLM knowledge base, and web searched knowledge. For example, it increased the number of cases with a MOI annotation by nearly threefold and those with defined zygosity by 4.7-fold. Inferring and interpreting such complex contextual clues by integrating both case data and the vast external knowledge base is prohibitive in time and technically difficult to conduct either manually or with the rigid rule-based bioinformatics methods as the author has attempted in the past.

Finally, the workflow facilitates fine-scale deep phenotyping. By disaggregating complex, abbreviated clinical descriptions into discrete HPO-mapped terms, the pipeline doubled the case by phenotype entries from 7480 to 14,000, with an average of 6.2 standardized phenotypes per patient. This granularity is essential for uncovering subtle genotype–phenotype–outcome correlations that could be missed in a less detailed and smaller dataset.

Compared with other GenAI-based tools targeting rare disease utilization, our framework is the only complete system for cohort-building which emphasizes practical utility by supporting both phenotype-to-HPO mapping and demographic data standardization, which achieved rapid construction of a large-scale and meta-analysis-ready LSS virtual cohort. In contrast, AutoMAxO focuses on treatment action dictionary of Medical Action Ontology [21], and RAG-HPO focused on improving HPO term mapping accuracy and reducing AI hallucinations [22], but they are not designed for cohort construction.

#### 4.1.2. Potential Limitations, Mitigations, and Future Directions

This GenAI pipeline has several limitations that need to be addressed. First, Gemini-2.5 Pro was anecdotally selected as the primary AI engine after LLM comparison based on its high accuracy in a single representative case, and future work should rigorously benchmark multiple LLMs. Second, we observed LLM “hallucinations”, especially in the assignment of HPO IDs from raw phenotype mapping, which were often inaccurate even when the corresponding HPO term names were correct. Our hybrid mitigation strategy utilized a human-in-the-loop QC process, where the AI-generated HPO mapping was validated against the source text and the HPO dictionary using a semantic similarity search tool. The expert review successfully corrected some questionable HPO mappings and ultimately achieved 91% accuracy for mapping phenotype terms.

GenAI inference may introduce two additional biases: First, as described above, 91% of GenAI-inferred HPO mappings were deemed correct after human-in-the-loop curation, leaving another 9% of these phenotype mappings being treated as implicitly “unknown” and being missed in the final cohort dataset. Another bias is the potential mapping errors not captured by the current human review and validation procedure, which may be mitigated by introducing multi-curator cross validation.

Source data bias is inherent to literature-based virtual cohort studies and warrants caution when interpreting these findings. A virtual cohort is a reflection of the published literature content plus curator selection preference. First, the cohort is subject to publication biases due to the inclusion criterion of studies with five or more cases which may introduce a selection bias against novel findings that are often presented in single case reports. More significantly, literature suffers from reporting bias that favors positive or novel disease and symptom findings over negative ones. The third is source data accessibility bias which is limited to freely accessible publications only.

As described above, source data bias and data loss during transformation workflow can lead to the absence of a reported phenotype. Such entries were regarded as “unknown” rather than a true “negative” finding, which may limit the capacity in defining complete genotype–phenotype correlations. These factors must be considered when interpreting the representativeness of cohort’s demographic, genetics, and clinical features. Expanding the source literature, improving curation and introducing mathematical inferences can help to reduce these biases.

The current framework provides a robust foundation for future enhancements. The first direction is optimizing the LLM prompt and improving the integration with the expert curation component. Another direction is fine-tuning a model trained specifically on rare disease corpora to improve HPO mapping accuracy and reduce manual curation. Another option is integrating 3rd party AI tools like RAG-HPO into our own system [22] and, lastly, integrating multi-curator cross-validation when resources and funding become available.

### 4.2. Clinical Insights from the LSS Virtual Cohort: A Comparative Analysis

#### 4.2.1. Age of Onset, Death, and Survival Time

The scale of our 1600-case virtual cohort provides unprecedented statistical power compared to previous population natural history studies. We found that LSS is predominantly a disease of early life, with 70.8% of cases present by age 2 as Congenital, Infantile, or Early Childhood onset. This is consistent with the findings of Lim et al. [14], who reported median onset age of 9 months and median age at death of 4.0 years in their 72-patient UK cohort, and the 96-case report from Sofou et al. [12], which also showed median age of disease onset at 7 months, median age at death at 2.4 years (range: 1 month–21 years), and the elapsed median time from disease onset to death of 1.8 years.

Analysis of 280 deceased patients revealed a median age at death of 2.08 years and a median survival time of 2.4 years post-onset. This confirms the devastatingly rapid disease progression and aligns with the poor prognosis reported [12,14], although the median survival time is slightly longer than reported by Sofou et al. [12].

#### 4.2.2. Phenotypic and Genetic Architectures of LSS Virtual Cohort and Comparison with Published Cohorts

The large-scale LSS virtual cohort confirms the genetic landscape with *SURF1*, *MT-ATP6*, and *MT-ND3* as the most frequently mutated genes, which is consistent with findings from both Lim et al.’s [14] and Sofou et al.’s [12] studies.

This meta-analysis constructed a comprehensive phenotypic landscape of LSS. The most prevalent clinical features are neurodevelopmental abnormalities (894 cases), abnormal brain morphology (963 cases), acidosis (794 cases), and decreased activity of mitochondrial respiratory chain (613 cases). Within these broad categories, the most common specific phenotypes were global developmental delay/regression, hypotonia, lactic acidosis, and characteristic bilateral lesions of the basal ganglia and brainstem.

These findings are consistent with other smaller cohort studies. Lim et al. identified poor function (mobility, self-care), extrapyramidal features (dystonia), and seizures as major contributors to disease burden in their cohort [14]. Our data corroborates these findings, with dystonia (154 cases) and seizures (351 cases) being prominent features. Sofou et al., in a 96-patient European cohort, highlighted ataxia, ophthalmoplegia, and cardiomyopathy as key features [12], all of which were frequently observed in our analysis (ataxia: 29 cases, ophthalmoplegia: 135 cases).

According to previous expert summaries for nuclear-LSS [5] and mtDNA-LSS [6], developmental delay affects 70% of LSS patients, followed by hypotonia at 65%, dystonia at 30~35%, ataxia at 30~50%, and spasticity at 40~50%. Muscle weakness affects 30% and 75% of cases in nuclear-LSS and mtDNA-LSS, respectively. These numbers are comparable to the most common phenotypes in the current meta-analysis, though we understand that due to the sparse clinical data across publications, the absolute percentages are expected to be lower in the virtual cohort.

#### 4.2.3. Over-Representation of Males Among X-Linked LSS Cases

The LSS virtual cohort has an over-representation of males (26 cases) compared to females (8 cases) for cases with X-linked inheritance. All the six LLS cases caused by *NDUFA1* variants are found in males in this cohort. This is supported by a separate 2009 report where both *NDUFA1* cases were also males [30]. The nine LLS cases caused by *PDHA1* variants also show a skewed male-to-female sex ratio of 7:2. These contrast with a previous mode of inheritance summary that speculated an equal sex ratio for LSS caused by *NDUFA1*, *HSD17B10*, or *PDHA1* [6], thus warranting further investigation. Together, our literature-derived large-scale virtual cohort led to new findings about MOI, sex ratio, and potential genetic etiology. Further expanding the cohort and improving data comprehensiveness through AI-enhanced curation will further contribute to molecular genetic analysis of rare diseases.

## 5. Conclusions

This study validated a scalable GenAI-driven framework for rapid case data standardization from sparse and highly heterogeneous data and rapidly created a unified LSS virtual cohort of over 1600 cases. The framework automates the most labor-intensive biocuration tasks, and the human-in-the-loop process combines domain expertise to generate a high-quality analysis-ready dataset with unprecedented cohort size, thus enabling well-powered survival analyses. Stratification by clinical and genetic features generated valuable insights and contributions to the mitochondrial disease field. We expect this framework to accelerate population-level study in Leigh Syndrome and serve as a template for other rare diseases, including mitochondrial diseases.

## Figures and Tables

**Table 1:** Characteristics of the top 10 included studies.

PMID	Cases	Publication Year	First Author	Journal/Book	Title and Reference Number
34428295	275	2021	Ratnaike TE	Nucleic Acids Res.	MitoPhen database: a human phenotype ontology-based approach to identify mitochondrial DNA diseases [16]
35094435	210	2022	Stenton SL	Ann. Neurol.	Leigh Syndrome: A Study of 209 Patients at the Beijing Children’s Hospital [13]
23829769	139	2013	Wedatilake Y	Orphanet J. Rare Dis.	*SURF1* deficiency: a multi-center natural history study [23]
27290639	114	2016	Pronicka E	J. Transl. Med.	New perspective in diagnostics of mitochondrial disorders: two years’ experience with whole-exome sequencing at a national pediatric center [24]
31967322	103	2020	Ogawa E	J. Inherit Metab. Dis.	Mortality of Japanese patients with Leigh syndrome: Effects of age at onset and genetic diagnosis [25]
34298071	100	2021	Imai-Okazaki A	Int. J. Cardiol.	Long-term prognosis and genetic background of cardiomyopathy in 223 pediatric mitochondrial disease patients [26]
28429146	69	2017	Ogawa E	J. Inherit Metab. Dis.	Clinical validity of biochemical and molecular analysis in diagnosing Leigh syndrome: a study of 106 Japanese patients [27]
30642647	66	2019	Imai-Okazaki A	Int. J. Cardiol.	Cardiomyopathy in children with mitochondrial disease: Prognosis and genetic background [28]
40716504	65	2025	Ahmadi ZA	Biochim. Biophys. Acta Mol. Basis Dis.	The genotype/phenotype conundrum of inherited mitochondrial disorders: Insights from a survey of mtDNA mutations associated with Leigh syndrome in complex I [29]
24731534	60	2014	Sofou K	Orphanet J. Rare Dis.	A multicenter study on Leigh syndrome: disease course and predictors of survival [11]

**Table 2:** Sex characteristics of the raw dataset.

Sex	Cases	Case Percentage	Known Death	Known Still Alive	Death%	Alive% at Last Record	Mean Age at Onset Years	Mean Survival Years
Female	731	45.50%	69	79	9.40%	10.80%	2.02	5.81
Male	874	54.50%	98	68	11.20%	7.80%	1.78	5.63
UNKNOWN	709	NA	NA	NA	NA	NA	0.75	3.75

**Table 3:** Top 10 rationales for GenAI phenotype mapping to HPO terms.

Rationale	Cases	Percentage in All Cases	Percentage in Case with Phenotype	Entries	Entry Percentage
Mapped from patient’s clinical description.	1718	74.24	78.95	12,339	81.74
Mapped from patient’s biochemical findings.	259	11.19	11.9	336	2.23
Mapped from patient’s clinical diagnosis.	143	6.18	6.57	143	0.95
Mapped from patient’s neuroimaging findings.	119	5.14	5.47	182	1.21
Mapped from patient’s clinical description (LS = Leigh Syndrome).	39	1.69	1.79	39	0.26
Mapped from the provided specific mitochondrial pathway defect.	39	1.69	1.79	39	0.26
Mapped from patient’s biochemical findings. CI is Complex I.	37	1.6	1.7	37	0.25
Mapped from neuroimaging findings (BG: basal ganglia).	34	1.47	1.56	34	0.23
Mapped from patient’s clinical description (DD).	34	1.47	1.56	50	0.33
Mapped from patient’s neuroimaging findings (BG).	34	1.47	1.56	34	0.23
Mapped from patient’s clinical description (MW).	31	1.34	1.42	31	0.21

**Table 4:** Case core data elements’ enrichment before and after GenAI data transformation.

Core Data Element Keys Inferred	Cases	Case Percentage	Case% Exclusively AI-Inferred *	Case% Exist in Raw Data **	Entries	Entry Percentage	Cases Before GenAI Curation, Remark
DIAGNOSIS	2307	99.7	64.7	35.3	2501	6.88	Not directly available before curation, Not directly comparable
GENE	1627	68.32	15.9	84.1	1622	4.46	Not directly available before curation, Not directly comparable
GENOTYPE (Zygosity)	832	35.96	56	44	838	2.3	176
MODE_OF_INHERITANCE (MOI)	1984	85.74	89.4	10.6	1994	5.48	685
PHENOTYPE	1916	93.99	94.9	5.1	15,095	41.49	Not directly available, Not directly comparable due to GenAI splitting and abbreviation expansion
VARIANT_cDNA	490	21.18	28	72	679	1.87	Not directly available before curation, Not directly comparable
VARIANT_mtDNA	734	31.72	17.4	82.6	737	2.03	654
VARIANT_PROTEIN	367	15.86	34.9	65.1	434	1.19	Not directly available before curation, Not directly comparable

Note: *: percentage of cases with the specific data element values inferred by AI but not explicitly stated in the raw case data. **: percentage of cases with specific data element values explicitly stated in the raw data.

**Table 6:** Summary of age of onset, death, and survival time.

Age at Onset in HPO Term	Cases	Case Percentage	Mean Age_at_Onset Years	Cases_with Death_or_Alive Info.	Mean Age_at_Death_or_Alive Last Record Years	Cases_with Survival_Years	Mean Survival Years
Antenatal onset	7	0.41	0	5	0.18	4	0.26
Congenital onset	83	4.86	0	52	4.21	26	4.62
Neonatal onset	88	5.16	0.01	40	2.86	29	2.92
Infantile onset	665	38.96	0.45	278	4.29	147	4.51
Childhood onset	565	33.1	2.07	202	7.95	64	9.43
Juvenile onset	209	12.24	7.43	69	8.52	5	7.22
Middle-age onset	10	0.59	53.33	2	23.5	1	1
Late onset	1	0.06	67	1	0.03	0	NA
Adult onset	9	0.53	19.57	0	NA	0	NA
Young adult onset	50	2.93	24.5	14	12.49	1	0.83
Age at Death in HPO Term	Cases	Case percentage	Mean Age_at_onset years	Cases_with Death_or_Alive info	Mean Age_at_death_or_Alive Last Record Years	Cases_with survival_years	Mean Survival Years
Neonatal death	23	1.35	NA	23	0.05	11	0.05
Death in infancy	128	7.5	NA	128	1.14	86	0.89
Death in childhood	142	8.32	NA	142	3.92	118	3.26
Death in adolescence	20	1.17	NA	20	11.58	13	11.32
Death in middle age	1	0.06	NA	1	47	1	1
Death in adulthood	4	0.23	NA	4	18.23	3	15.42
Death in early adulthood	7	0.41	NA	7	23.49	5	21.43

**Table 7:** Mitochondrial biochemical function group vs. case age and survival time.

Gene Function Group	Death Status	Cases	Case Percentage	Age at Onset Years	Mean Survival Years	Remark
Complex I	Died	47	6.68	0.64	3.7	Longer survival in DEATH group
Complex IV	Died	75	10.65	0.98	3.57	
Complex V	Died	14	1.99	1.65	1.77	Later onset, with shorter survival
Mitochondrial translation	Died	26	3.69	0.23	2.99	Earliest onset
mtDNA depletion and/or multiple mtDNA deletions	Died	3	0.43	0.49	1.18	
Fission	Died	1	0.14	0.17	1.33	
Detoxification	Died	1	0.14	0.67	1.33	
OXPHOS ± PDHc enzyme deficiency/Disorders of mitochondrial toxicity	Died	3	0.43	0.25	3.05	
Complex I	Alive	76	10.8	2.02	7.3	Later onset, longer survival- at last alive age
Complex IV	Alive	58	8.24	1.4	3.86	
Complex V	Alive	31	4.4	1.52	4.88	
Mitochondrial translation	Alive	5	0.71	1.57	3.67	
OXPHOS ± PDHc enzyme deficiency	Alive	2	0.28	0.71	0.12	
Mitochondrial stability, fission, clearance by mitophagy	Alive	1	0.14	2	2.25	
OXPHOS ± PDHc enzyme deficiency/Disorders of mitochondrial toxicity	Alive	14	1.99	0.6	8.17	Earlier onset, longest survival- at last alive age
Proteostasis/Defect of mitochondrial protein quality control	Alive	1	0.14	1	5.92	
Pyruvate dehydrogenase complex	Alive	1	0.14	0.58	0.42	
Biotin/Thiamine	Alive	4	0.57	0.28	6.09	

## Data Availability

The complete, harmonized case-level dataset generated and analyzed during this study is publicly available at the Leigh Syndrome Spectrum (LSS) Virtual Cohort Case Browser (Figure S1, URL: https://mseqdr.org/clinical/lss.php, accessed on 25 January 2026). The summaries of the dataset are also provided in the main text and Supplementary Materials of this article. All source data were derived from public PubMed resources, and the specific publications are cited in Supplementary Table S3. Further inquiries regarding the data or methodology can be directed to the corresponding author upon reasonable request.

## Supplementary Materials

The following supporting information can be downloaded at: https://www.mdpi.com/article/10.3390/biology15040334/s1, Supplemental Material S1. Prompt–Ai4Mito-Age; Figure S1: Leigh Syndrome Spectrum (LSS) Virtual Cohort Case Browser; Table S1: GenAI-Transformed Core Data Elements for Clinical and Demographic Characteristics from Published Raw Column Names. Table S2: Harmonized Data Dictionary for Clinical, Genomic, and Demographic Fields. Table S3: PubMed sources with five or more cases for the virtual cohort. Table S4: Consolidated Phenotypes with at Least 200 Cases Including the Child Terms in HPO Ontology Based on the 2314 Raw Cases. Table S5: Gemini-2.5-pro. Table S6: ChatGPT-5. Table S7: qwen3-30B-A3B. Table S8: Model: Phi4-mini. References [5,11,13,14,16,23,24,25,26,27,28,29,31,32,33,34,35,36,37,38,39,40,41,42,43,44,45,46,47,48,49,50,51,52,53,54] are cited in the Table S3.
